# Assessment of the Aging of the Brown Adipose Tissue by ^1^ ^8^F-FDG PET/CT Imaging in the Progeria Mouse Model Lmna^−/−^

**DOI:** 10.1155/2018/8327089

**Published:** 2018-07-11

**Authors:** Zhengjie Wang, Xiaolong Xu, Yi Liu, Yongheng Gao, Fei Kang, Baohua Liu, Jing Wang

**Affiliations:** ^**1**^ Department of Nuclear Medicine, Xijing Hospital, Fourth Military Medical University, No. 127 West Changle Road, Xi'an 710032, China; ^2^Department of Orthopaedics, Xijing Hospital, Fourth Military Medical University, No. 127 West Changle Road, Xi'an 710032, China; ^3^Medical Research Center (MRC), Shenzhen University Health Science Center, Shenzhen 518060, China; ^4^Department of Biochemistry and Molecular Biology, Shenzhen University Health Science Center, Shenzhen 518060, China; ^5^Guangdong Key Laboratory for Genome Stability and Human Disease Prevention, Shenzhen University Health Science Center, Shenzhen 518060, China

## Abstract

Brown adipose tissue (BAT) is an important energy metabolic organ that is highly implicated in obesity, type 2 diabetes, and atherosclerosis. Aging is one of the most important determinants of BAT activity. In this study, we used ^18^F-FDG PET/CT imaging to assess BAT aging in Lmna^−/−^ mice. The maximum standardized uptake value (SUV_Max_) of the BAT was measured, and the target/nontarget (T/NT) values of BAT were calculated. The transcription and the protein expression levels of the uncoupling protein 1 (UCP1), beta3-adrenergic receptor (*β*3-AR), and the PR domain-containing 16 (PRDM16) were measured by quantitative real-time polymerase chain reaction (RT-PCR) and Western blotting or immunohistochemical analysis. Apoptosis and cell senescence rates in the BAT of WT and Lmna^−/−^ mice were determined by terminal deoxynucleotidyl transferase dUTP nick end labeling (TUNEL) and by CDKN2A/p16INK4a immunohistochemical staining, respectively. At 14 weeks of age, the BAT SUV_Max_ and the expression levels of UCP1, *β*3-AR, and PRDM16 in Lmna^−/−^ mice were significantly reduced relative to WT mice. At the same time, the number of p16INK4a and TUNEL positively stained cells (%) increased in Lmna^−/−^ mice. Collectively, our results indicate that the aging characteristics and the aging regulatory mechanism in the BAT of Lmna^−/−^ mice can mimic the normal BAT aging process.

## 1. Introduction

Aging has been defined as the age-related deterioration of physiological functions of an organism. The main characteristic of the aging process is the gradual decline of the functions of the organ system. Brown adipose tissue (BAT) is an adipose organ which is responsible for maintaining the core temperature in small mammals and in newborn humans [1]. The functional status of BAT is closely related to obesity, type 2 diabetes, and atherosclerosis [2, 3].

Aging is one of the most important determinants of BAT activity [4]. Yoneshiro et al. has reported that cold-induced brown fat activity is greatly decreased with aging [5]. Therefore, it is important to study the relationship between BAT functional status and aging. Since 1996, many long-term studies have investigated the relationship between BAT function and aging by assessing BAT-related indicators [6–8]. These indicators included cold-induced heat generation, cold tolerance, body temperature, and other macrophysiological parameters as well as molecular biomarkers, such as uncoupling protein 1 (UCP1), PPAR-g coactivator 1a (PGC1a), PR domain-containing 16 (PRDM16), and beta3-adrenergic receptor (beta3-AR) [6–8]. However, there are still some concerns regarding the above studies. Firstly, the mice of the research model used in the above studies have normal aging rate, with a life duration of up to 32 weeks, so research takes long time, and the degree of aging is difficult to define [9]; secondly, the research method, the macroscopic physiological indicators, or microscopic molecular markers used in the above studies are only indirect indicators of the metabolic activity in the BAT and often do not accurately reflect the functional status of BAT. Thus, a visualized imaging method and a unified premature aging model are necessary to investigate the metabolic profile changes and the regulation mechanism of BAT aging.

Since 2003, the presence of adult BAT was confirmed by ^18^F-FDG PET scanning [10]. Currently, ^18^F-FDG PET/CT imaging is considered to be the “gold standard” in basic research and clinical studies for assessing BAT function *in vivo* [10–12] and has been widely used in the basics and clinical studies of BAT. The functional status of BAT can be directly calculated from the glucose metabolism activity levels [12].

Hutchinson–Gilford progeria syndrome (HGPS) is caused by a mutation in the *Lmna* gene, and the mouse model of HGPS used in this study is a widely used aging model in basic research studies [13–15]. Abbreviated as Lmna^−/−^ mice, these types of mice have demonstrated classical presenile manifestations in multiple organs, such as skin, liver, and kidneys [16, 17]. However, clinical evidence has shown that HGPS patients are free of cognitive deterioration in the central nervous system [18], which may be attributed to the inhibitory effect of the microRNA *miR-9* on progeria [19]. Therefore, progerin-induced aging is very likely to be organ specific. In order to assess the applicability of the Lmna^−/−^ mice model in BAT-related aging studies, we addressed the potential implication of the BAT function of Lmna^−/−^ mice in premature senility. Furthermore, whether the levels of BAT function measured by ^18^F-FDG PET/CT uptake levels are related to the levels of the aforementioned molecular aging markers would provide additional information about the feasibility of using this mouse model in BAT-related aging research.

In this study, ^18^F-FDG PET/CT imaging technique was used to qualitatively and quantitatively analyze the relationship between ^18^F-FDG PET/CT imaging and BAT aging in Lmna^−/−^ mice. The relationship between the levels of the BAT-related molecular markers, UCP1 and *β*3-AR, and ^18^F-FDG uptake was examined. In addition, the mechanism of BAT function in Lmna^−/−^ mice was also studied.

## 2. Materials and Methods

### 2.1. Animal Preparation and ^18^F-FDG Micro-PET/CT Imaging

Lmna^−/−^ mice were obtained from the Medical Laboratory Animal Department of The Fourth Military Medical University, and experiments were performed in accordance with the Animal Experimental Ethics Committee of The Fourth Military Medical University approved protocols. Aging mice were identified by PCR (Supplementary Figure 1). Mice were cold-treated under fasting conditions for a duration of 4 hours before they received one dose of ^18^F-FDG [20], ensuring that the mice were fasting but with free access to water. 200–300 uCi of ^18^F-FDG in 150 *µ*l of saline was intraperitoneally injected into each mouse. The mice were maintained in the cold for one additional hour after FDG injection and then scanned with a small-animal-dedicated micro-PET/CT system. The rectal temperature was measured using a PET/CT accessory. Once anesthesia was induced, the animals were moved onto mice beds with their heads resting within a conical face mask that continuously delivered 2% isoflurane at a flow rate of 1.5 L/min. An electric heating pad, provided by the small-animal PET/CT system, was placed under the animals to help maintain their body temperature. PET/CT data were acquired for 600 s for each mouse under continuous anesthesia. All PET/CT images were processed and analyzed using the Nucline nanoScan software (Mediso). For semiquantitative analysis, three-dimensional (3D) regions of interest were carefully drawn and manually adjusted, according to the CT images, over the borders of the BAT on the small-animal PET images of each mouse. Three-dimensional round regions of interest were delineated on the lung as a nontarget (NT) reference. Tracer uptake rates by the BAT and the lung tissue were quantified as the maximum standardized uptake values (SUV_Max_) using the formula: SUV_Max_ = maximum tissue activity concentration (Bq/mL)/injected dose (Bq) × body weight (g). The average uptake ratio (T/NT) of the mean BAT (T) over the mean lung tissue (NT) uptake was calculated and compared.

### 2.2. Quantitative Real-Time Reverse Transcription PCR

After the mice were sacrificed, total RNA was isolated from BAT using the Total RNA Kit I (Omega Bio-Tek, USA) according to the manufacturer's guidelines. Single-stranded cDNA was synthesized from total RNA with the PrimeScript™ RT Master Mix (TaKaRa, China). Real-time RT-PCR for each target was performed with the SYBR® Premix Ex Taq II polymerase (TaKaRa). The thermal cycling conditions were set as follows: preheating for 30 seconds at 94°C, 40 cycles of denaturation (10 seconds at 94°C), annealing (30 seconds at 60°C), and elongation (20 seconds at 72°C). For each sample, the mRNA levels were normalized to the mRNA levels of the internal control, *β*-actin. The pairs of primers used are listed in Table 1.

### 2.3. Western Blot Analysis

After the mice were sacrificed, protein was extracted; the total protein concentration in the samples was determined using the bicinchoninic acid (BCA; Boster, China) method and by adding 5 × loading buffer and then heating at 100°C for 5 min. 40 *μ*g of protein from each sample was loaded onto 10% sodium dodecyl sulfate-polyacrylamide 1 mm gels and transferred onto nitrocellulose (NC) membranes. To detect the target proteins, we blocked the membranes for 2 h with 5% bovine serum albumin (BSA; Boster, Hubei, China) at 4°C. Then, the membranes were incubated overnight at 4°C with anti-*β*3-AR (1 : 1000, Abcam, USA) antibodies or anti-UCP1 (1 : 1000, Cell Signaling Technology, USA) specifically recognizing the target proteins. The membranes were subsequently washed with Tris-buffered saline containing Tween-20 (TBST) for 5 min, and the process was repeated five times. Then, the membranes were incubated for 1 h at room temperature with secondary antibody. The membrane was washed five times in TBST for 5 min. Blots were exposed and developed using the enhanced chemiluminescence (ECL) method. The images were captured and analyzed by the ImageJ software.

### 2.4. Immunohistochemistry Analysis

The BAT was harvested from freshly sacrificed mice and fixed in 10% formalin. Formalin-fixed, paraffin-embedded tissue blocks were serially cut into 3 mm thick sections, which were dewaxed in xylene and rehydrated through a graded series of ethanol solutions. After 3 washes in PBS, heat-induced antigen was retrieved in 0.01 M citric acid buffer (pH 6.0) and autoclaved for 5 min at 120°C. Nonspecific binding sites were blocked through preincubation with normal bovine serum albumin for 30 min. Slices were washed 3 times in PBS for 5 min. These tissue sections were then incubated with anti-*β*3-AR antibodies (1 : 100; Abcam), anti-UCP1 antibodies (1 : 50; Cell Signaling Technology), PRDM16 (1 : 100; Abcam), or p16INK4a (1 : 500; Abcam), which were diluted in PBST buffer containing 4% BSA and 2.5% Triton X-100. Then, the tissue slices were incubated with horseradish peroxidase-conjugated anti-rabbit IgG (1 : 1000; EarthOx). In all the sections, the positively labeled cells were visualized using 3,3-diaminobenzidine tetrahydrochloride (Shanghai Sangon) as a chromogen and were counterstained with hematoxylin. Quantification of the immunostaining was performed by digital image analysis with the Image-Pro Plus 6.0 software (Media Cybernetics). A total of 3 fields of view per slice were selected for imaging hot-spot areas (400x objective lens), and 5 slices from each animal were acquired. In each imaging field, the integrated optical density (IOD) and the area of interest (AOI) of all the positively stained area were measured. The IOD was used to evaluate the area and intensity of the positive staining. The mean density (IOD/AOI) represents the concentration of a specific protein per unit area.

### 2.5. Evaluation of Cell Apoptosis in the Brown Adipose Tissue

Tissue apoptosis was assessed by performing the TUNEL staining (Beyotime, Beijing, China) assay on the sample slices, according to the manufacturer's protocol. Briefly, after dewaxing and hydration, sections were incubated with TUNEL reaction mixture. Nuclei were stained using DAPI. Afterwards, slides were observed using a fluorescence microscope (400x; TE-2000U, Nikon, Japan). For each staining, a total of 3 sections per group were observed.

### 2.6. Statistical Analysis

All values are expressed as mean ±SEM. Statistical analysis was performed using GraphPad Prism software. The results of PET imaging over time were compared between WT and Lmna^−/−^ mice using the two-way ANOVA with post hoc test. The differences between the two groups were determined by Student's *t*-test. Differences with *P* < 0.05 were considered statistically significant.

## 3. Results

### 3.1. Effects of Age on the Metabolic Activity of BAT in Mice

**Figure 1:**
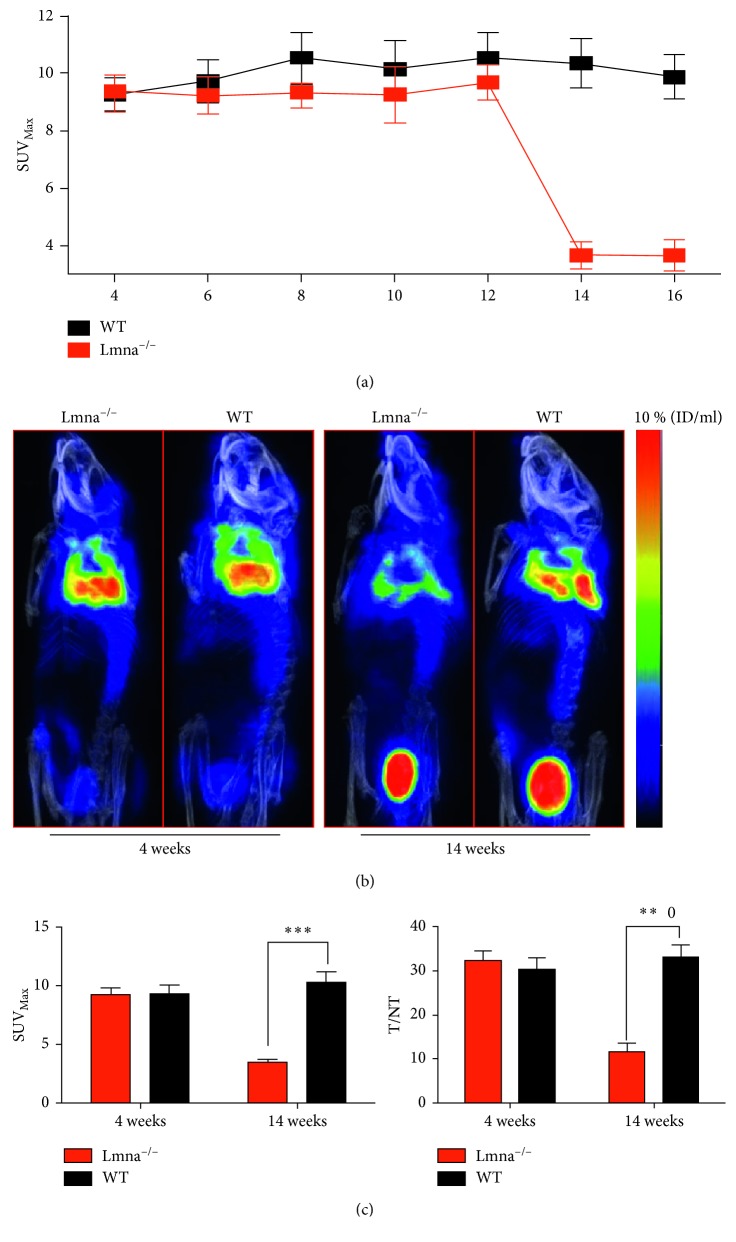
PET images of the BAT from Lmna^−/−^ and wild-type (WT) mice. (a) The changes of SUV_Max_ in the BAT from 4 to 16 weeks of age. (b) Lmna^−/−^ and WT mice PET/CT images at 4 weeks and 14 weeks of age. (c) Quantification of SUV_Max_ and T/NT (target/nontarget) values in the BAT of Lmna^−/−^ and WT mice at 4 and 14 weeks. Lung tissue was defined as the NT reference. ^∗^ *P* < 0.05; ^∗∗^ *P* < 0.01; ^∗∗∗^ *P* < 0.001.

To study the effect of age on Lmna^−/−^ and WT mice, PET/CT imaging was performed from 4 weeks on Lmna^−/−^ and WT mice, once every two weeks and for 16 weeks. As shown in Figure 1(a), there was a significant difference in PET imaging results over time between WT and Lmna^−/−^ mice (*P* < 0.05). From 4 to 12 weeks, there was no significant difference in the SUV_Max_ of the BAT between Lmna^−/−^ and WT mice, indicating that FDG uptake during the first 12 weeks was similar between the two mice strains. As shown in Figures 1(b) and 1(c), at the fourth week of PET imaging, there was no difference in the levels of ^18^F-FDG uptake between Lmna^−/−^ and WT mice, but at 14 weeks of age, ^18^F-FDG uptake in the BAT of Lmna^−/−^ mice was significantly lower than that in WT mice. Statistically, the SUV_Max_ value and the T/NT ratio of BAT did not differ significantly between Lmna^−/−^ and WT mice at 4 weeks of age (SUV_Max_ value: 9.260 ± 0.571 versus 9.373 ± 0.709, *P*=0.9069; T/NT ratio: 32.27 ± 2.278 versus 30.41 ± 2.411, *P*=0.6223). At 14 weeks of age, both measurements in the Lmna^−/−^ mice were significantly lower than those in WT mice (SUV_Max_ value: 3.673 ± 0.4613 versus 10.34 ± 0.8663, *P*=0.0003 T/NT ratio: 11.61 ± 1.975 versus 33.16 ± 2.687, *P*=0.0030). These findings confirm that the BAT ^18^F-FDG uptake in Lmna^−/−^ mice decreased significantly at 14 weeks of age.

### 3.2. Effects of Age on BAT in Mice

**Figure 2:**
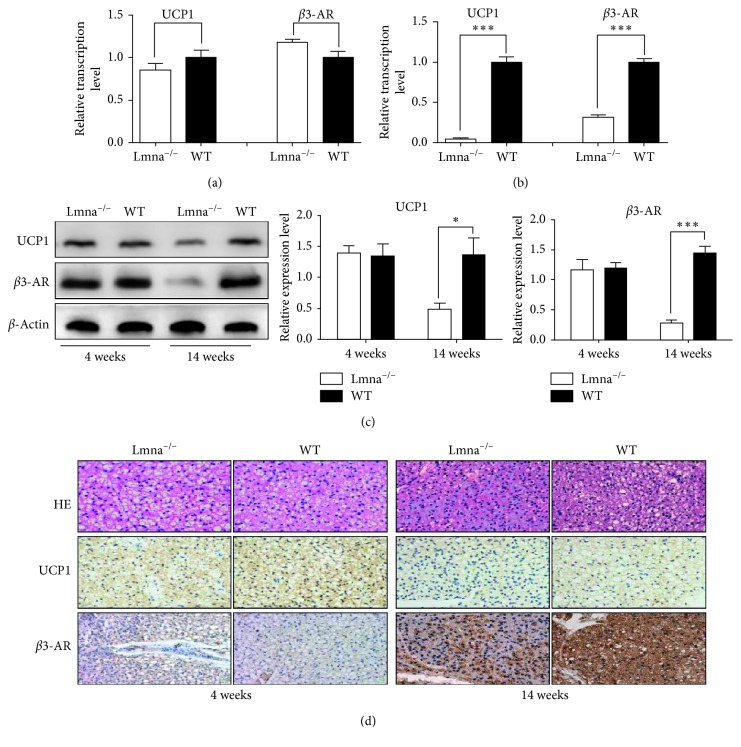
Analysis of beta3-AR and UCP1 levels in the BAT of Lmna^−/−^ and WT mice. (a, b) Relative transcription levels of UCP1 and *β*3-AR in Lmna^−/−^ and WT mice at 4 and 14 weeks of age. (c) Western blot analysis for UCP1, beta3-AR, and *β*-actin and semiquantitative analysis of the expression levels of UCP1 and *β*3-AR. (d) BAT hematoxylin and eosin staining and immunohistochemical analysis of beta3-AR and UCP1. ^∗^ *P* < 0.05; ^∗∗^ *P* < 0.001; ^∗∗∗^ *P* < 0.001.

To investigate the causes of the reduction in BAT ^18^F-FDG uptake in Lmna^−/−^ mice, the mRNA expression levels of UCP1 and beta3-AR were assessed. As shown in Figure 2(b), UCP1 and beta3-AR protein levels in Lmna^−/−^ mice were significantly lower than those in WT mice at 14 weeks of age (UCP1: 0.0474 ± 0.0089 versus 1.000 ± 0.0666, *P*=0.0001; beta3-AR: 3143 ± 0.0329 versus 1.000 ± 0.0445, *P*=0.0002). At 4 weeks of age, as shown in Figure 2(c), there was no difference in the mRNA expression levels of UCP1 and beta3-AR between Lmna^−/−^ and WT mice (UCP1: 1.403 ± 0.1213 versus 1.342 ± 0.1959, *P*=0.8026; beta3-AR: 1.200 ± 0.9090 versus 1.170 ± 0.1703, *P*=0.8223), while at 14 weeks of age, the expression of UCP1 and beta3-AR was decreased (UCP1: 0.4854 ± 0.1012 versus 1.367 ± 0.2706, *P*=0.0380; beta3-AR: 0.2830 ± 0.0497 versus 1.445 ± 0.1189, *P*=0.0008). As shown in Figure 2(d), immunohistochemical analysis results were consistent with Western blot results. These findings show that both the protein and mRNA expression levels of UCP1 and beta3-AR, the molecular markers of BAT activity, are reduced in Lmna^−/−^ mice, which is consistent with the aging characteristics of the BAT of healthy mice [21].

**Figure 3:**
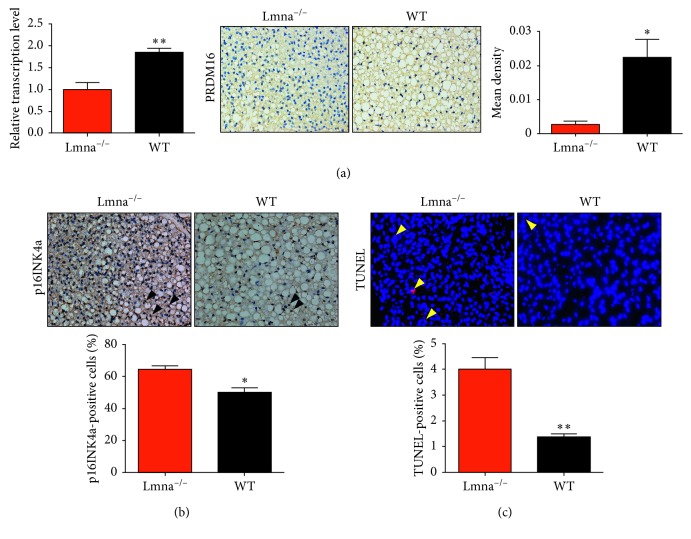
Analysis of the PRDM16 expression levels, and cell senescence and apoptosis rates in the BAT of Lmna^−/−^ and WT mice. (a) Relative transcription levels and immunohistochemical analysis of PRDM16 in Lmna^−/−^ and WT mice at 14 weeks of age. (b, c) Images of p16INK4a immunostaining (black, open arrowheads) and TUNEL (yellow, open arrowheads) in the BAT of Lmna^−/−^ and WT mice and quantification of positive labeling for p16INK4a and TUNEL. ^∗^ *P* < 0.05; ^∗∗^ *P* < 0.01.

To study the causes of BAT dysfunction, the expression of PRDM16 was also assessed. In addition, CDKN2A/p16INK4a immunostaining was performed to assess cellular senescence [22], while apoptotic cells in the BAT were quantified by performing terminal deoxynucleotidyl transferase dUTP nick end labeling (TUNEL). As shown in Figure 3, both the protein and mRNA levels of PRDM16 were decreased at the age of 14 weeks in Lmna^−/−^ mice (1.000 ± 0.1582 versus 1.850 ± 0.0945, *P*=0.0099). A significant increase in the number of p16INK4a-expressing cells was observed in the BAT of Lmna^−/−^ mice (64.33 ± 2.333% versus 50.33 ± 2.603, *P*=0.0161). Likewise, the number of TUNEL-positive cells in the BAT of Lmna^−/−^ mice was higher than that in WT mice (4.0 ± 0.45% versus 1.36 ± 0.1202, *P*=0.0049).

### 3.3. Effects of Age on the Body Weight of Mice

**Figure 4:**
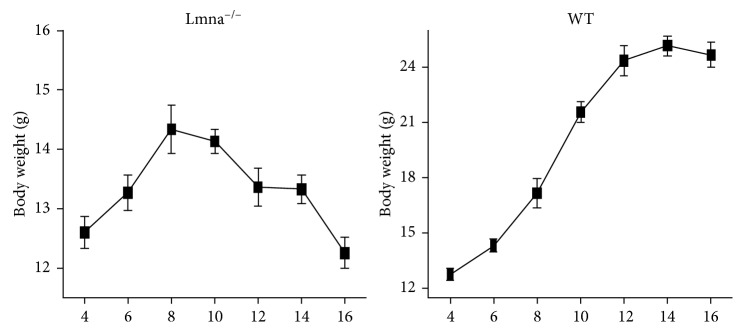
Changes in the body weight during aging in Lmna^−/−^ and WT mice.

As shown in Figure 4, the body weight of Lmna^−/−^ mice began to decline at the 10th week of age, while the weight of wild mice continued to increase, showing that, at that stage, the Lmna^−/−^ mice started to lose weight, indicating a decline in the metabolic state of their body.

## 4. Discussion

In this study, we examined the changes of BAT function in relation to age and investigated the relationship between ^18^F-FDG PET/CT imaging and aging in the BAT of Lmna^−/−^ mice. The mechanism of BAT dysfunction was also explored. Our study led to these major findings: Firstly, the BAT function of the Lmna^−/−^ mice significantly decreased, and the uptake of ^18^F-FDG was reduced at the age of 14 weeks. Secondly, the mechanism of BAT aging in Lmna^−/−^ mice is associated with a reduction in the number of brown adipocytes and an increase in the number of senescent and apoptotic cells. Thirdly, the state of the body of Lmna^−/−^ mice began to decline at 10 weeks of age, which is earlier than the observed ^18^F-FDG uptake decrease.

Aging is an irreversible natural process. It is projected that the world senior and geriatric population will reach 2.1 billion by 2050 [23]. Aging is the primary risk factor for major human pathologies, including cancer, diabetes, cardiovascular disorders, neurodegenerative diseases [24], and is one of the most important determinants of BAT activity in all animals, from rodents to humans [25]. Since BAT activity has important physiological significance, it is of great importance to study the mechanism of BAT aging. An aging model for the study of BAT is necessary. However, there were no reported aging models suitable for BAT studies until recently [26, 27]. In our study, we found that the Lmna^−/−^ mice BAT displayed age-related dysfunction characteristics, which would be beneficial for BAT aging research. Utilizing Lmna^−/−^ mice for studying BAT aging could resolve the problem of long study periods, inconsistent determination of aging degree, and other model-related issues.

Lmna^−/−^ mice BAT dysfunction can mimic normal mice BAT aging changes. The changes in the BAT of normal mice were predominantly associated with the reduction in the levels of *β*3-AR and UCP1 [28]. We found that the expression of *β*3-AR and UCP1 in Lmna^−/−^ mice decreased in a short time and the BAT exhibited age-related dysfunction features. The reduction in the ^18^F-FDG uptake in the BAT of Lmna^−/−^ mice was closely related to the decreased expression of *β*3-AR and UCP1. Exposure to cold has been known to stimulate the sympathetic nervous system via binding of the neurotransmitter norepinephrine to the *β*3-AR [29]. *β*3-AR stimulation has been reported to increase glucose uptake in BAT [30] through induction of transcription and *de novo* synthesis of glucose transporter molecule 1 (GLUT1) [31]. Likewise, we also found that Lmna^−/−^ mice GLUT1 transcription levels were significantly lower than those in WT mice (Supplementary Figure 2). Under cold stimulation, *β*3-AR not only increases glucose transport, but also promotes UCP1 expression and activity [32]. Although Hankir MK et al. proposed that BAT ^18^F-FDG uptake and UCP1-mediated heat production can be dissociated [33], most studies suggest that UCP1 is necessary for norepinephrine-induced glucose utilization in BAT [30]. Therefore, the reduction in the levels of UCP1 in Lmna^−/−^ mice was likely responsible for the reduction of ^18^F-FDG uptake in the BAT.

The mechanism of BAT dysfunction in Lmna^−/−^ mice also simulates the mechanism of BAT aging in normal mice [34]. The premature aging of the HGPS mouse model is attributed to a point mutation in position 1824 of the *Lmna* gene, which results in the conversion of cytosine to thymine in the encoded protein. This mutation creates an abnormal splice donor site, which produces a truncated protein (progerin), lacking residues 607–656 of prelamin A but retaining the C-terminal CAAX box, a target for prenylation [15]. Progerin cannot be detached from the nuclear membrane and ultimately damages the structure and function of the nucleus [35]. Progerin also participates in the normal process of aging [36, 37]. Therefore, HGPS mice exhibit an accelerated rate of a subset of pathological changes that together drive a faster aging process [24, 38]. BAT aging in Lmna^−/−^ mice mimicked the physiological aging of BAT in two aspects. Firstly, brown fat cell formation was reduced. This reduction was consistent with the decrease in the gene expression levels of PRDM16 in Lmna^−/−^ mice, which acts as a transcriptional coregulator that controls the development of brown adipocytes in BAT [39]. Secondly, the increased apoptosis and senescence rate of brown adipose tissue in Lmna^−/−^ mice could result in a reduction in the number of brown adipocytes, an observation which also mimics the physiological aging of BAT [40].

Aging is a systemic change, which in Lmna^−/−^ mice causes an early decrease in the body weight that can be observed at the 10th week of age. The mice of the HGPS model are lean [41] and display myopathic disease [42], which leads to body weight reduction earlier than the observed BAT ^18^F-FDG uptake decrease, indicating that in Lmna^−/−^ mice, the decline in the metabolic state of the body initiates before BAT aging.

However, the results of our study have some limitations. Firstly, it should be pointed out that the aging mechanism triggered by the Lmna^−/−^ phenotype may not be the only reason for the altered glucose metabolism function and apoptosis process of the BAT. Progeria and other Lmna-linked diseases may also influence the ^18^F-FDG uptake in the BAT. For example, progerin has been reported to have cytotoxicity effects and to induce mitochondrial damage [43]. In addition, it has been described that Lmna^−/−^ mice are likely to display slow heart rates and Lmna-linked hypoglycemia [17], which could also inhibit ^18^F-FDG uptake. More research is needed to investigate the magnitude of the effect of progeria and other Lmna-linked diseases on the degeneration of BAT. Secondly, we studied the aging of BAT in Lmna^−/−^ mice from the perspective of glucose metabolism. ^18^F-FDG is currently considered the ‘gold standard' for the assessment of BAT metabolic activity, but it cannot discriminate between oxidative and nonoxidative BAT glucose metabolism [44, 45]. In addition to ^18^F-FDG, other tracers are still available to be used for studying metabolic activity changes in the aging of BAT [46]. Therefore, for a more comprehensive assessment of the BAT metabolic activity, the application of other tracers is also required.

## 5. Conclusion

Lmna^−/−^ mice are an ideal model for studying BAT aging. The ^18^F-FDG uptake and the levels of BAT-related molecular markers were decreased in Lmna^−/−^ mice at 14 weeks of age. The aging characteristics and the aging mechanism of BAT in Lmna^−/−^ mice can mimic the normal process of BAT aging.

## Figures and Tables

**Table 1:** Primers used in quantitative RT-PCR experiments.

Primer	Primer sequence (5′-3′)
*β*-actin-S	GTGACGTTGACATCCGTAAAGA
*β*-actin-A	GTAACAGTCCGCCTAGAAGCAC
*β*3-AR-S	CCTTCCGTCGTCTTCTGTGTAG
*β*3-AR-A	CTGTTGAGCGGTGGACTCTG
Ucp1-S	ACAGTAAATGGCAGGGGACG
Ucp1-A	CACGGGGACCTACAATGCTT
GLUT1-S	ACGCCCCCCAGAAGGTTAT
GLUT1-A	GCGTGGTGAGTGTGGTGGAT
PRDM16-S	ATGGGATCCATGAAGAACGGT
PRDM16-A	CACGTCTACGGTGAACGGAA

## Supplementary Materials

Supplementary Materials Supplementary Figure 1: identification of Lmna-/- mice. (a) The photograph of the three kinds of mice at 14 weeks of age. Het, heterozygote; (b) Lmna-/-mice were identified by PCR and Western blot. Progerin, the truncated type of Lamin A. Supplementary Figure 2: relative transcription levels of GLUT1 in the BAT of Lmna^−/−^ and WT mice. ^∗∗∗^ *P* < 0.001
