# Supplementary material for: Assessment of the Aging of the Brown Adipose Tissue by 1 8F-FDG PET/CT Imaging in the Progeria Mouse Model Lmna−/−
Source: Contrast Media Mol Imaging. 2018 Jul 11;2018:8327089. doi: 10.1155/2018/8327089 (PMC6079616; doi:10.1155/2018/8327089)
Supplement: Supplementary Materials — Supplementary Figure 1: identification of Lmna-/- mice. (a) The photograph of the three kinds of mice at 14 weeks of age. Het, heterozygote; (b) Lmna-/-mice were identified by PCR and Western blot. Progerin, the truncated type of Lamin A. Supplementary Figure 2: relative transcription levels of GLUT1 in the BAT of Lmna−/− and WT mice. ∗∗∗ P < 0.001 [file 8327089.f1.pdf]

SUPPLEMENTARY MATERIAL

Assessment of the aging of the brown adipose tissue by ^{18}F-FDG PET/CT imaging in the progeria mouse model Lmna$^{-/-}$

Zhengjie Wang[1, #], Xiaolong Xu[2, #], Yi Liu[1, #], Yongheng Gao[1], Fei Kang[1, *], Baohua Liu[3,4,5 *], Jing Wang[1, *]

Materials and Methods

The primers of the Lamin A were synthesized from the Takara. Forward primer: CTATTGCATGCTTCTCCTCAG; Reverse primer: TGAGCGCAGGTTGTACTCAG. The PCR conditions to amplify the Lamin A were set as follows: pre-heating for 5 minutes at 95°C, and 40 cycles of denaturation (30 seconds at 95°C), annealing (60 seconds at 59°C) and elongation (30 seconds at 72°C), and 5 minutes at 72°C. The Lamin A/C antibody was purchased from the Abcam(EPR4068).

FIGURE LEGENDS

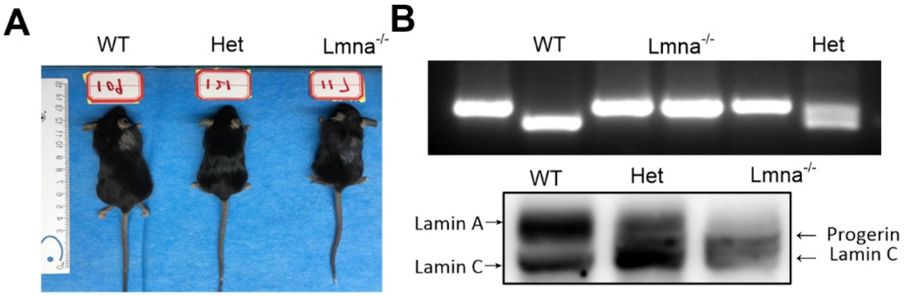

Supplementary figure 1. Identification of Lmna$^{-/-}$ mice. (A) The photograph of the three kinds of mice at 14 weeks age. Het, Heterozygote; (B) Lmna$^{-/-}$ mice were identified by PCR and western blot. Progerin, the truncated type of Lamin A.

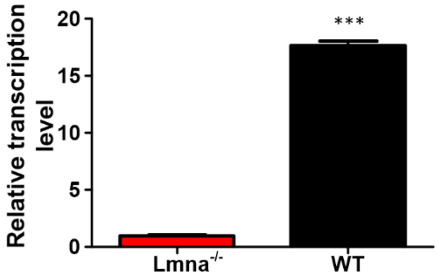

Supplementary figure 2. Relative transcription levels of GLUT1 in the BAT of Lmna$^{-/-}$ and WT mice. *** $P < 0.001$.
